# Growth and shell hardness of the apple snail *Pomacea flagellata* Say, 1829, reared at three calcium concentrations

**DOI:** 10.7717/peerj.14840

**Published:** 2023-02-09

**Authors:** Alberto De Jesús-Navarrete, Derian J. C. Aguilar Sanchez, María C. Ortiz-Hernández

**Affiliations:** 1 Department of Systematic and Aquatic Ecology, El Colegio de la Frontera Sur, Chetumal, Quintana Roo, Mexico; 2 Sistematica y Ecología Acuática, El Colegio de la Frontera Sur, Chetumal, Quintana Roo, México; 3 Department of Sustainability Sciences, El Colegio de la Frontera Sur, Chetumal, Quintana Roo, Mexico

**Keywords:** Aquaculture, Freshwater, Gastropods, Shell, Growth, Mollusck

## Abstract

The snail *Pomacea flagellata* inhabits aquatic systems with high calcium concentration and it is important to food webs; unfortunately, its natural populations are decreasing due to overfishing and habitat destruction. Here we tested the effect of three water calcium concentrations on the growth and hardness of snail shells in triplicate recirculation culture systems for 12 weeks. In each culture, 100 juvenile snails were seeded at constant density and fed with balanced tilapia feed. Thirty snails were randomly collected every 15 days and measured in length and total weight. The size, weight, and shell hardness of the snails for the 500 mg/L calcium treatment were significantly higher than the mean size of the snails in the other treatments (300 mg/L and 243.33 mg/L). The calcium supply in the culture promotes growth and allows the snails to produce healthier and stronger shells, in addition to improving their growth rate, which is important for the management of the species.

## Introduction

The apple snail (*Pomacea flagellata* Say, 1829), known locally as the “chivita” snail, is a gastropod native to tropical waters of America (Naranjo-García, 2003; Vázquez-Silva et al., 2011). Despite being a prolific organism and resistant to various environmental conditions, the number of organisms has decreased in the natural environment, mainly due to overfishing, since it is a local food; now, due to increased tourism, it is offered as a snack in the local restaurants. Also, the modification and destruction of their habitat due to the growth of tourism infrastructure has contributed to the decrease of these snails (Iriarte-Rodríguez & Mendoza-Carranza, 2007; De Jesús-Navarrete et al., 2019).

In the Yucatan peninsula, the apple snail is distributed mainly in the Bacalar Lake, an aquatic system that feeds on groundwater, with a high calcium condition (325 mg/L), which allows the presence of microbialites; a diverse microbial communities that precipitate carbonates, silicates and sulfate minerals (Yanez-Montalvo et al., 2020) and mollusk communities such as the apple snail *Pomacea flagellata* and the bivalve *Mytilopsis sallei* Rècluz, 1849 (Gischler et al., 2011; De Jesús-Navarrete et al., 2019). Due to the decrease in the natural populations of snails in the Bacalar Lake, an aquaculture protocol is carried out for the first time under controlled conditions in the laboratory, which will allow the raising of snails in the laboratory to repopulate the lagoon and produce organisms for human consumption, these management strategies would contribute to the conservation of the species. These gastropods can adapt quickly to controlled systems, and it is possible to maintain them in laboratory conditions (*i.e*., in aquaria or plastic containers). They also have characteristics that facilitate their management and culture; for example, these snails can reproduce throughout the year, grow quickly, and tolerate low oxygen concentrations because they have a pulmonary sac and a siphon that allows the snail to breathe atmospheric air. These features provide the potential for aquaculture that uses low cost and simple equipment (Amador-del Ángel et al., 2006; Brito-Manzano et al., 2007; Benavides-Linares, Chacón-Piche & Portillo-Segovia, 2012; Coelho, Calado & Dinis, 2012). They require a pH between 6.5 and 8.0, and their shells will begin to erode at pH values ≤5.0. In cultures of *Pomacea*, there is a high requirement of calcium salts for shell formation because the calcium concentration in water can directly affect the development of organisms (Hunter & Lull, 1977; Hunter, 1990; Glass & Darby, 2009) in the case of freshwater pulmonated gastropods most of the shell calcium is actively taken up from the water, rather than the diary snail diet (Van der Borght & Van Puymbroeck, 1966; Greenaway, 1971).

Muño-Rivera (1987), who worked with the diets of *P. flagellata*, mentioned the possibility of abrasion and fracture of the snail shells during the cultivation period, possibly due to the low concentration or absence of calcium carbonates in the water; however, without taking measurements of carbonate concentration, this assumption could not be confirmed.

Currently, there are several studies relating snail growth with different types of diets in freshwater snails (Muño-Rivera, 1987; Ozaeta-Zetina, 2002; Ruiz-Ramírez, Espinosa-Chávez & Martinez-Jerónimo, 2005; Iriarte-Rodríguez & Mendoza-Carranza, 2007; De Jesús-Carrillo, 2014) and over the importance of calcium content in different diet types and shell growth (Rundle et al., 2004; Bukowski & Auld, 2014). The formation of shells of snails is a complex and poorly understood process from a mechanical point of view; but the importance of two ions, Ca^2+^ and HCO^−^_3_, are well studied. Freshwaters Ca is extracted from the environment against a concentration gradient (Greenaway, 1971; Ponder, Lindberg & Ponder, 2019). However, there are few studies evaluating the effect that different water concentrations of calcium have on the growth and resistance of the shell (Hunter, 1990; España-Herrera & Sánchez-Castañeda, 1995; Rundle et al., 2004; Glass & Darby, 2009; AL-Asadi, 2011; Markoglou et al., 2011).

The aim of this study was to determine the effect of different calcium concentrations in water on the growth and hardness of the snail *Pomacea flagellata* shell under experimental laboratory conditions, keeping in mind that increased calcium availability (saturated conditions) will lead to greater growth and a more robust shell in snails.

## Materials and Methods

This research was conducted at the El Colegio de la Frontera Sur, Mollusks Laboratory during spring 2015. The snails were reared in a 96 m^2^ (8 × 12 m) area roofed with zinc sheeting, with natural photoperiod and ambient temperature. We used nine plastic vats (210 L each one) aligned and supplied with water flow driven by independent submersible pumps at each calcium concentration. To achieve our objective, the experimental design considered three triplicate treatments, with different calcium concentrations (*n* = 9): A = similar to the calcium conditions of Bacalar Lake (300 mg/L Ca), natural habitat of *Pomacea flagellata*, B = the concentration of calcium equivalent to 500 mg/L Ca, and Control (C) = Tap water, no calcium added (equivalent to 243.33 mg/L). Water samples were collected from Bacalar Lake to determine the Ca content to reproduce the water conditions of the lagoon in treatment A (300 mg/L Ca). In addition, a sample of tap water was taken in the laboratory to determine the concentration of Ca (243.33 mg/L).

The vats of each treatment were filled with tap water and allowed to sit for 48 h to remove excess chlorine; then, calcium sulfate dihydrate was added to create the desired calcium (Ca) levels. We select this compound (Baker Analyzed; CaSO_4_ * 2 H_2_O, Lot No. E01702, 101% pure. Molar mass 172.17 g/mol) for its easy solubility. During the experiment, every week, the calcium concentrations in the vats of the Chemistry Laboratory ECOSUR were determined by atomic absorption spectrophotometry (NMX-AA-051-SCFI-2001) and, if necessary, the concentrations were maintained adding calcium sulfate dihydrate.

Snails used for the experiment were obtained from snail eggs masses collected in Bacalar Lake. Six egg masses collected from the emergent vegetation in Bacalar Lake which was placed in a box lined with plastic bubbles and taken to the laboratory where egg masses were placed in tanks with water over a steel mesh until their hatched (Benavides-Linares, Chacón-Piche & Portillo-Segovia, 2012; De Jesús-Carrillo, 2014). Snails were selected for the growth experiment from different egg masses that hatched at the same time, and 900 snails were randomly chosen and placed to the vats.

For the growth experiment, nine vats recirculation systems were used, and there were three calcium treatments. A total of 900 juvenile snails were used, 100 in each vat, all juvenile snails that were seeded in the vats were measured and weighed to know their initial size. A constant density was maintained by replacing dead snails with similar-size snails to balance the number of individuals at each calcium concentration. New juveniles were marked with red nail polish (which does not inhibit growth or kill snails) to differentiate the aggregated snails from the originally seeded snails.

Juveniles were fed with balanced food for tilapia: 35% protein, fiber, 5.0%, calcium 3.2% and phosphorus 0.9%, in a proportional amount to 15% of the total biomass of snails by vat for the first 5 weeks, 10% for weeks 6 to 10, and 7.5% for week 11 onwards; the adjusted portion was based on biometric data that was collected to obtain a daily portion supply amount (De Jesús-Carrillo, 2014). The concentration of calcium in the diet was the same in all treatments and it was within the optimum concentration for the growth of snails (Dalesman & Lukowiak, 2013) Water temperature and pH were recorded weekly using an Oakton potentiometer model 510 pH meter “All-in-One” pH/Temp electrode, 110/220 VAC.

A sample of 30 snails was randomly selected in each vat, and the size (total length) and weight were measured every 15 days after hatching. During the first few weeks, juveniles were measured with an ocular micrometer calibrated in a stereoscope and posteriorly with a digital caliper to determine the size in mm and weighed on an analytical balance HR-200 with 0.0001 g resolution. The experiment had a duration of 12 weeks (*n* = 1,890 data). In the case of weight measurements, the selected snails were placed on a sieve (500 microns of mesh light) to remove water as much as possible before being weighed, to avoid weighing errors due to variation in the water content of the snails. To analyze the shell variation with time, the measurement data were grouped (*n* = 1,890), and a simple growth equation was calculated. To compare the differences between average shell length and average weight of snails among the three concentrations of Ca in the culture, an ANCOVA model was applied (Zuur, Leno & Smith, 2007) with shell length as response variable (SL) and time (t), as the quantitative variable, and treatment (Ca^+^) as factor, all at three levels. Additionally, the relationships between length and weight were evaluated using the weight-length relationship (P = a. L^b^), which was linearized, and it was analyzed with an ANOVA (R Development Core Team, 2008).

To determine the differences between the strength of the shell, (crushed shell weight kg^−1^), the shells of the snails belonging to the three treatments were compared. To carry out this test, 10 snails from each calcium concentration were selected, and at the end of the experiment, snails were immersed in hot water at a temperature of 80 °C for 15 min to allow muscular-visceral softening, facilitating its removal, and then the empty shells were exposed to the sun.

The crushing of the shells was carried out using a prefabricated device consisting of a metal can, which was lying on a wooden platform. Individual shells were placed between the center of the platform and the can, the opening of the shell was placed in contact with the platform, water was added to the can to add the weight needed to break the shell, and the amount of water was determined (subtracting the weight of the can) and recorded in kilograms. After using the Kolmogorov-Smirnov test to assess normality of variance, one-way ANOVA was used to compare the shell crush weight of juveniles between treatments. ANOVA results were followed by Tukey’s HSD *post hoc* test. All statistical tests had a significance level of 5% (α = 0.05), and analyses were performed with the statistical package R (R Development Core Team, 2008).

## Results

**Figure 1:**
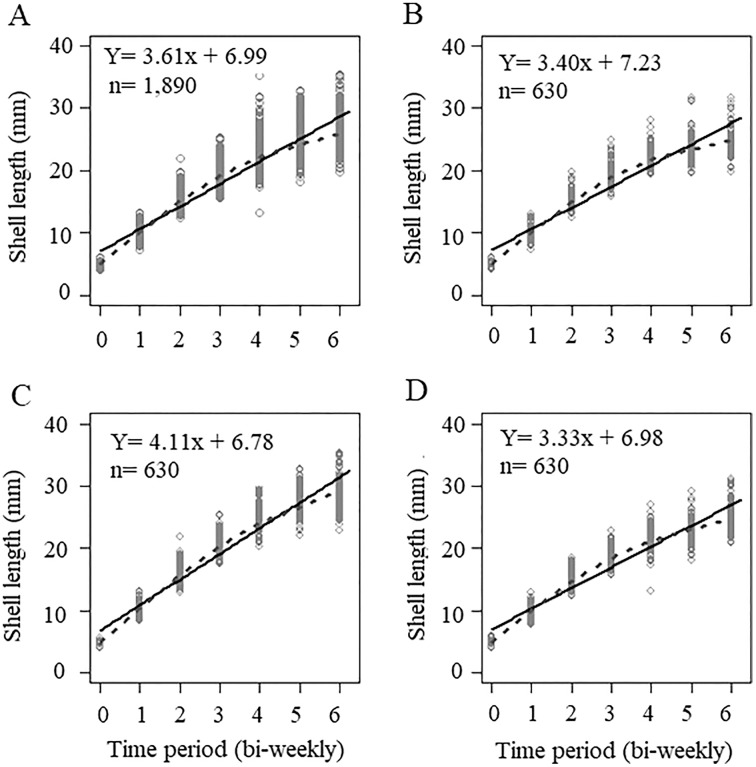
Growth curve based on averages considering shell sizes at three levels. (A) Not considering calcium concentration; (B) growth curve at treatment A = 300 mg l^−1^; (C) growth at 500 mg l^−1^; and (D) growth at 243.33 mg l^−1^. Continuous line = Lineal regression, dotted line = smoothing model.

The initial sizes and weights of juvenile snails, coming from different egg masses and being randomly selected, were not the same in each treatment. For treatment A, the average seeding size was 5.05 ± 0.39 mm and an average weight of 0.033 ± 0.009 g, for treatment B, the average seeding size was 4.76 ± 0.34 mm, with an average weight of 0.023 ± 0.004 g, and treatment C had an average size of 4.91 ± 0.38 mm, and an average weight of 0.028 ± 0.0089 g. The initial sizes of treatments A and C were higher than treatment B (Table S1). The growth curve of all the organisms measured during the experiment (*n* = 1,890) and at each concentration is shown in Fig. 1. Because the organisms were seeded in different initial size, the curve does not intercept the origin; rather, it intercepts the average value of the initial size of the snails. When we considered the global data, the slope of the line was 3.61, which indicated the growth rate of the organisms over time, the smoothing model represented by the dotted line shows an adjust with some asymptotic tendency, however, the lineal model is a good representation of the data (Fig. 1A). For the concentration in treatment A (300 mg/L), the growth rate was 3.40, (Fig. 1B), which was less than that value obtained when considering the three interaction levels (3.61), we observed that the smoothing curve follows the same tendency that the lineal model. The concentration of treatment B (500 mg/L) was significantly different and had a slope of 4.11 (*p* < 0.5, Table 1), which showed that this calcium concentration produced faster growth in snails (Fig. 1C), even when the initial size and weight at treatment B was lower than A and C treatments. The control concentration (C = 243.33 mg/L) showed the lowest growth rate, with a value of 3.33 (Fig. 1D) in these three cases the smoothing model has a lineal behavior. ANCOVA showed that time and calcium concentration had significant effects on snail length (*p* < 0.5), and the ANOVA applied to ANCOVA indicated that there were significant differences between snail length with respect to time and concentration (*p* < 0.05) (Table 1).

**Table 1:** ANCOVA analysis between shell length and time. The calcium concentration is considered independent variable. Estimations of interception and slope are included.

[Ca] level	Interception, *a*	Slope, *b*	Comments
All concentrations	6.99320 $\approx$ 6.99	3.61394 $\approx$ 3.61	All data, *n* = 1,890; without considering Ca concentrations
Concentration A	7.22563 $\approx$ 7.23	3.39706 $\approx$ 3.40	Only considering data of calcium concentration 300 mg/L; *n* = 630
Concentration B	6.77492 $\approx$ 6.78	4.11149 $\approx$ 4.11	Only considering data of calcium concentration 500 mg/L; *n* = 630
Concentration C	6.97904 $\approx$ 6.98	3.33435 $\approx$ 3.33	Only considering data of calcium concentration 243.33 mg/L; *n* = 630

The effect of Ca concentration on growth, measured as the size and weight of individuals, can also be evaluated in terms of the weight-length relationship. The highest value of exponent b of the weight-length relationship was recorded in calcium concentration B (b = 3.06), followed by the value corresponding to concentration C (b = 3.05) and then that of concentration A (b = 3.01) (Table 2).

**Table 2:** Shell crush resistence. ANOVA results of the weight-to-length ratio and the calcium concentration as a factor; includes estimates of the different intercept values and the slope of linearized equations using the logarithm transformation. *Significative differences (*p* < 0.05).

Calcium [Ca]	Intercept, *a*	Slope, *b*	Comments
All treatments	Exp (−8.519553) 0.00019953	3.0381 $\approx$ 3.04	All data, *n* = 1,890; not including calcium concentration.
Concentration A	Exp (−8.41397) 0.00022175	3.0097 $\approx$ 3.01	Only considering data of calcium concentration 300 mg/L; *n* = 630
Concentration B	Exp (−8.62426) 0.00017969	3.0583 $\approx$ 3.06*	Only considering data of calcium concentration 500 mg/L; *n* = 630
Concentration C	Exp (−8.54300) 0.00019491	3.0543 $\approx$ 3.05	Only considering data of calcium concentration 243.33 mg/L; *n* = 630

**Figure 2:**
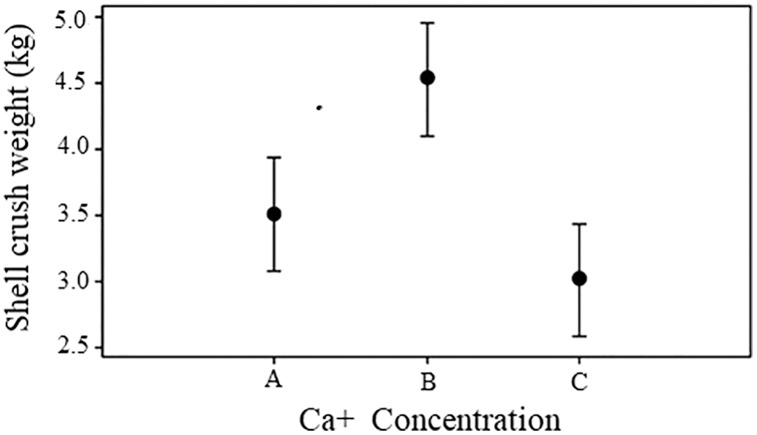
Shell crush resistance. Shell crush strength for snails reared in three concentrations of calcium in water. Bars represents standard deviation. A = 300 mg l^−1^, B = 500 mg l^−1^ and C = 243.33 mg l^−1^.

At the end of the experiment, the shells of snails grew at a Ca concentration of 500 mg/L and showed greater resistance than the snail shells from the control conditions and the concentrations of 300 mg/L (Fs = 6.84, *p* = 0.004). However, only the difference in the shells observed between snails reared at concentrations of 500 mg/L and the control was significant (Tukey HSD = 1.02 *p* > 0.10) (Fig. 2).

The water temperature throughout the culture time ranged from 24 °C to 29 °C; the temperature during week 6 and in weeks 9–12 was above the optimal range, while the temperature in weeks 2–5 remained within the optimum range (24–26 °C), pH values were over 6.0 during weeks 1 to 8 varying from 8 to 6.14, lower pH values were measured at week 9, with pH = 5.4 for the of 300 mg/L calcium concentration, pH = 4.86 for the 500 mg/L concentration, and pH = 5.25 for the control concentration. It was observed that the dead snails in this period had little damaged shells. In summary, during weeks 1 to 8, the pH remained within the optimal range, while for weeks 9–12, the pH readings indicated slightly more acidic conditions and were below the optimal range (6.5–8).

## Discussion

The Bacalar Lake in Quintana Roo, Mexico, is characterized by a high concentration of calcium in its waters and harbors the presence of microbialites, but also an important population of mollusks, among them the “chivita” *Pomacea flagellata* snail (Yanez-Montalvo et al., 2020). The results obtained in this experiment confirm that calcium variations can influence shell growth and resistance, as previously found by Rundle et al. (2004) for *Lymnaea stagnalis* Linnaeus, 1758, and by Glass & Darby (2009) for *P. paludosa* Say, 1829 in Florida.

The experiment carried out by España-Herrera & Sánchez-Castañeda (1995) showed that the growth of *Pomacea* sp. based on size and weight was enhanced by the addition of calcium salts. In a 4-month period, the snails reached average sizes of 14.8 mm for 125 mg/L, 16 mm for 250 mg/L, and 18.7 mm for 500 mg/L, with a lettuce-based diet. These results are below those reported in this work since we obtained mean sizes of 25 mm for the concentration of 243.33 mg/L, 25.22 mm for the concentration of 300 mg/L, and 28.79 mm for the concentration of 500 mg/L at the end of the 3-month culture period. The difference in size gain is probably since tilapia feed contains 3.2% calcium, which is the minimum required percentage that has been reported in the case of other freshwater gastropods (Dalesman & Lukowiak, 2013). Glass & Darby (2009) showed that the growth of *P. paludosa* Say, 1829 was affected by low calcium concentrations; these authors raised snails at Ca concentrations of 10, 20, 40, 80, and 100 mg/L for a period of 6 weeks. In their experiments, the snails showed slow growth in low calcium concentrations, while high concentrations favored the growth of these organisms. The snail’s calcium is absorbed mainly by food intake, in our case, percentage of calcium was 3.2%, however, a portion of the snail’s calcium is provided by the concentration of dissolved calcium in the water, which allows snails to grow faster and build thicker shells (Van der Borght & Van Puymbroeck, 1966; Greenaway, 1971). This fact has been shown for *Planorbella trivolvis* Say, 1817 (Hunter, 1990), *Lymnaea stagnalis* Linnaeus, 1758 (Rundle et al., 2004), *Lymnaea peregra* Müller, 1774 and *Planorbarius corneus* Linnaeus, 1758 (White et al., 2007).

The measurements of length and weight are related to growth and production in mollusks (King, 2007). In our experiment it is confirmed that the greater availability of calcium (500 mg/L) led to an exponent of the equation of length-weight higher (b = 3.06), however, the lowest calcium concentration (243.33 mg/L) was not substantially different since the value of the exponent was (b = 3.05) and greater than the calcium concentration of 300 mg/L, The differences between these values of the exponent, could be explained by the variances in the sowing snail sizes in each concentration, or due to a better efficiency of the juveniles raised with tap water compared with treatment A.

The diet experiment carried out by De Jesús-Carrillo (2014) involved the use of tap water in a concentration similar to that reported in the control concentration of this work (243.33 mg/L of Ca); at 16 weeks, the following growth metrics were reported: 11.77 mm for individuals fed “chaya” (*Cnidoscolus aconitifolius* Mill I. M. Johnstl), 11.83 mm for individuals fed spinach, and 14.44 mm for individuals fed a commercial shrimp feed. These results are below those reported in this work, since in our culture, we obtained an average size of 28.79 mm for individuals fed commercial food for tilapia. It is evident that the contribution of protein-rich diets such as shrimp feed and food for tilapia generates greater growth in snails. When combining high protein diets with calcium-rich water concentrations, better snail production can be obtained in a shorter time.

The apple snail *Pomacea* can survive in a pH range from 4 to 10 without significantly mortality (Bianchini et al., 2022); however, values below six cause problems in the shell, fissures, decalcification, anorexia, reproductive problems and decreases in growth, while values higher than 10 also produce negatively affect the organism (Byers et al., 2013), for example, in *Planorbella trivolvis*, with low pH and low calcium concentration, snails had lower growth rates and reduced fecundity (Hunter, 1990), in this sense a higher concentration of calcium in water can promote a better growing in snails and hardest shells (Greenaway, 1971; Ewald et al., 2009).

In their experiments on calcium and pH with *P. paludosa* Say, 1829, Glass & Darby (2009) reported that snails exposed to concentrations of 5.7 mg/L Ca and pH = 6.1 developed thinner shells and had problems with feeding, decalcification, while snails exposed to concentrations of 78 mg/L Ca and pH = 7.6 developed thicker shells with statistically significant differences. This result is compatible with our results since, when we compare the resistance to crushing snail shells subjected to different concentrations, we find that the snails with the most resistant shells were those raised with the highest calcium concentration; in wild snail populations, in an ecological approach a thinner shell could represent a higher predation rate (Bukowski & Auld, 2014).

The pH and temperature values remained similar between the three concentrations during the growing period, which meant that they had no effect on the differences in sizes and weights between our treatments. The results showed that the pH value was related to temperature, and the weeks with the highest temperature during this experiment had the lowest pH values, which coincided with the results reported by De Jesús-Carrillo (2014), who found a relationship between the average values of pH and temperature. Glass & Darby (2009) reported that temperature and pH were important variables for the successful development of aquacultures of different species. In snails, these factors can affect shell growth and formation, and low pH is associated with slow growth and low calcium fixation in the shell (Hunter, 1990; Rundle et al., 2004; Seuffert & Martin, 2013) but at low pH values, apparently snails use environmental calcium as a buffer source (Hunter, 1990; Ewald et al., 2009).

## Conclusions

In conclusion, snails reared at a concentration of 500 mg/L showed better growth and built stronger shells than snails raised at low concentrations. The supply of calcium in the *Pomacea* culture improves growth and allows snails to produce healthier and stronger shells, and it could improve the reproduction on the clutch of egg masses. The correct growth and development of snails is linked to several factors that must be considered when designing a culture method for these organisms. It is recommended that, in species management strategies, mainly in the case of repopulation with cultivated organisms, water with calcium concentrations greater than 300 mg/L is used. From the ecological point of view, snails raised at a higher concentration of calcium will produce organisms with more resistant shells that would avoid predation.

## Supplemental Information

10.7717/peerj.14840/supp-1 Supplemental Information 1 Raw data experiment snails growth at different calcium concentration. Length and weight growth data during the experiment

## Additional Information and Declarations

### Data Availability

The following information was supplied regarding data availability: The raw data is available in the Supplemental Files.
